# A retrospective classification of diagnoses in terms of DSM-5 for patients included in randomized controlled trials of *Ginkgo biloba* extract EGb 761^®^

**DOI:** 10.1007/s00406-015-0632-y

**Published:** 2015-08-13

**Authors:** Robert Hoerr, Michael Zaudig

**Affiliations:** Clinical Research Department, Dr. Willmar Schwabe GmbH & Co. KG, Willmar-Schwabe-Str. 4, 76227 Karlsruhe, Germany; Klinik Windach, Schuetzenstr. 100, 86949 Windach, Germany

**Keywords:** DSM-5, Diagnosis, Dementia, Mild cognitive impairment, Ginkgo biloba, EGb 761^®^

## Abstract

When the early trials of *Ginkgo biloba* extract EGb 761^®^ were conducted, different terms were used to denote ageing-associated neurocognitive disorders. With the fifth edition of the Diagnostic and Statistical Manual of Mental Disorders (DSM-5), a taxonomy covering dementia and pre-dementia stages of such disorders became available. DSM-5 diagnostic criteria for neurocognitive disorders (NCDs) were applied to patients with any type of ageing-associated cognitive impairment, including dementia, enrolled in randomized controlled clinical trials of EGb 761^®^, taking into account the reported inclusion and exclusion criteria and patient characteristics at baseline. For 23 of 31 trials (74 %), the inclusion diagnoses could be classified as NCD in accordance with DSM-5. Thirteen trials enrolled patients with major NCD, four trials enrolled patients with mild NCD and six trials enrolled patients with NCD, who could not be classified unambiguously as having mild or major NCD. Although various terms were formerly used for neurocognitive disorders, the patients enrolled in the majority of clinical trials with EGb 761^®^ could be classified retrospectively using modern DSM-5 diagnostic criteria.

**Electronic supplementary material:**

The online version of this article (doi:10.1007/s00406-015-0632-y) contains supplementary material, which is available to authorized users.

## Introduction

When the earliest clinical trials of the defined, quantified *Ginkgo biloba* extract EGb 761^®^ and some other drugs, then called “nootropics”, were conducted during the 1970s and 1980s, there was no widely accepted diagnostic term, let alone consensus diagnostic criteria, for what was later called “ageing-associated cognitive decline” (AACD) [1], “cognitive impairment no dementia” (CIND) [2] or “mild cognitive impairment” (MCI) [3, 4]. For cognitive impairment in the elderly, vaguely defined terms were often used, e.g.: “organic brain syndrome”, “cerebral insufficiency”, “impairment of cerebral performance”, “Hirnleistungsstörungen” (German), “troubles du vieillissement cérébral” (French) or, when associated with cerebrovascular disease, “cerebrovascular insufficiency”. The DSM-III [5] already provided a description and diagnostic criteria for dementia and research diagnostic criteria for Alzheimer’s disease (AD) [6] and vascular dementia (VaD) [7] followed soon. The DSM criteria have been revised repeatedly (DSM-III-R [8]; DSM-IV [9]). In addition to the DSM criteria, the diagnostic criteria of the International Classification of Diseases (ICD-10) [10] were also used in clinical trials. A comparison of these sets of criteria is provided in Table 1. In spite of the availability of diagnostic criteria and related nomenclature, there was continued reluctance among European clinicians to assign a diagnosis of dementia to a patient, as dementia was perceived as stigmatizing. Hence, descriptive terms that were neither precisely defined nor part of the thesaurus of a systematic classification of diseases were often used for conditions along the continuum of MCI and dementia until the end of the millennium. As a consequence, it was difficult to gauge whether patients enrolled in different clinical trials in fact had different disorders or whether different terms for the same disorder were being used in different publications.

**Table 1:** Comparison of diagnostic criteria for major neurocognitive disorder or dementia across classification systems

DSM-5	DSM-IV	DSM-III-R	DSM-III	ICD-10	NINCDS-ADRDA	NINDS-AIREN
Major NCD	Dementia	Dementia	Dementia	Dementia	Dementia	Dementia
A. Evidence of significant cognitive decline from a previous level of performance …	A. Development of … B. … represent a significant decline from a previous level of functioning	*From the explanatory text (“loss of intellectual abilities”) it can be concluded that cognitive impairment is to be understood as a consequence of cognitive decline*	A. Loss of intellectual abilities …	G1. Evidence of … (G1.1) Decline in … (G1.2) Decline in … deterioration from previously higher level of performance should be established	Dementia … Progressive worsening of …	1. Dementia defined by cognitive decline from a previously higher level of functioning and manifested by …
A. … in one or more cognitive domains … based on:	A. … multiple cognitive deficits manifested by *both*	*A. and B. must be met, i.e. multiple deficits required*	*B and C must be met, i.e. multiple deficits required*	G1. … each of the following:	Deficits in two or more areas of cognition	*As specified below: memory and two or more domains*
A1. Concern of the individual, a knowledgeable informant or the clinician *and*						
	A1. Memory impairment	A. Demonstrable evidence of impairment in short- and long-term memory	B. Memory impairment	(G1.1) … memory	… memory …	… impairment of memory …
A2. Substantial impairment in cognitive performance, documented by standardized neuropsychological testing or another quantified clinical assessment	A2. one or more of the following disturbances: (a) aphasia, (b) apraxia, (c) agnosia, (d) disturbance in executive functioning	B. At least one of the following: (1) impairment in abstract thinking, (2) impaired judgment, (3) other disturbances of higher cortical function, such as aphasia, apraxia, agnosia, constructional difficulty …	C. At least one of the following: (1) impairment of abstract thinking, (2) impaired judgment, (3) other disturbances of higher cortical function, such as aphasia, apraxia, agnosia, constructional difficulty …	(G1.2) … other cognitive abilities characterized by deterioration in judgement and thinking, such as planning and organizing, and in the general processing of information; … objectively verified by obtaining a reliable history, supplemented, if possible, by neuropsychological testing or quantified cognitive assessments	… and other cognitive functions … established by clinical examination and documented by MMSE, Blessed Dementia Scale or similar examination and confirmed by neuropsychological tests	… and of two or more cognitive domains (orientation, attention, language, visuospatial functions, executive functions, motor control, praxis), preferably established by clinical examination and documented by neuropsychological testing
		B. … (4) personality change	C. … (4) personality change	G3. Decline in emotional control or motivation, change in social behaviour, manifest as at least one of the following: (1) emotional lability, (2) irritability, (3) apathy, (4) coarsening of social behaviour		
B. Cognitive deficits interfere with independence in everyday activities	B. Cognitive deficits in A1 and A2 each cause significant impairment in social or occupational functioning and …	C. Disturbance in A and B significantly interferes with work or usual social activities or relationships with others	A. … of sufficient severity to interfere with social or occupational functioning	Mild: … interfere with everyday activities; Moderate: … serious handicap to independent living;	*Presence of dementia required, which—by definition—includes interference of deficits with activities of daily living*	Deficits should be severe enough to interfere with activities of daily living not due to physical effects of stroke alone
C. Cognitive deficits do not occur exclusively in the context of a delirium	E/D. Deficits do not occur exclusively during the course of a delirium	D. Not occurring exclusively during the course of delirium	D. State of consciousness not clouded (does not meet criteria for delirium or intoxication)	G2. Preserved awareness of the environment (i.e. absence of clouding of consciousness) during a period long enough to unequivocally demonstrate G1	No disturbance of consciousness	Exclusion criteria are disturbance of consciousness, delirium, psychosis, severe aphasia, or major sensorimotor impairment precluding neuropsychological testing
D. Cognitive deficits are not better explained by another mental disorder	F. Disturbance is not better accounted for by another Axis I disorder (only in AD criteria)	E. Either (1) evidence of a specific organic factor judged to be aetiologically related to the disturbance or (2) aetiological organic factor can be presumed if the disturbance cannot be accounted for by any nonorganic mental disorder	E. Either (1) evidence of a specific organic factor judged to be aetiologically related to the disturbance or (2) organic factor can be presumed if conditions other than organic mental disorder have been reasonably excluded and if the behavioural change represents cognitive impairment in a variety of areas	G4. For a confident clinical diagnosis, G1 should have been present for at least 6 months	Absence of systemic disorders or other brain diseases that in and of themselves could account for the progressive deficits in memory and cognition Onset between ages 40 and 90, most often after age 65	Excluded are systemic disorders or other brain diseases that in and of themselves could account for deficits in memory and cognition

Until recently, in contrast to dementia, the pre-dementia stages of neurocognitive disorders were poorly accounted for by disease classifications. Whereas the various types of dementia were classified as distinct entities with specific aetiologies in earlier editions of the DSM and the International Classification of Diseases (ICD), a heterogeneous collection of conditions between healthy ageing and dementia was subsumed under the term MCI. It was only in 2007 that Dubois and colleagues [11] took a “longitudinal” perspective when preparing a revision of the NINCDS-ADRDA criteria for Alzheimer’s disease. They proposed prodromal or MCI stages corresponding to each aetiologically defined dementia disorder. Similarly, with the change from DSM-IV to DSM-5, the classification was refined to cover the whole range of disorders from clear-cut MCI to full-blown dementia under the category “neurocognitive disorders” (NCDs) [12]. There is now provision (although not a strict requirement) for aetiological differentiation within both “mild neurocognitive disorder” (representing the MCI stage) and “major neurocognitive disorder” (representing dementia). From an ethical point of view, it is noteworthy that the new nomenclature no longer uses the term “dementia”, which has been perceived as pejorative and stigmatizing [13].

The question addressed by this research is to what extent the inclusion diagnoses and criteria applied in clinical trials of *G. biloba* extract EGb 761^®^ fit within the new DSM-5 taxonomy. Using published information and, as far as needed and available, unpublished data, we retrospectively classified the patients enrolled in randomized controlled trials of EGb 761^®^ in terms of DSM-5 diagnostic categories.

## Methods

Published papers on all randomized controlled trials of EGb 761^®^ in cognition-related ailments and disorders were retrieved. These were identified by a comprehensive literature search in the context of the call for scientific data on *G. biloba* for assessment by the Committee on Herbal Medicinal Products (HMPC) of the European Medicines Agency in October 2011. All available information about diagnostic terms, diagnostic criteria, inclusion and exclusion criteria, presence and actual severity of cognitive and functional impairment at baseline was retrieved at study level and checked against the DSM-5 criteria for major NCD and mild NCD and their aetiological sub-classes. If an unambiguous classification was not possible from the published information, the original clinical trial reports were retrieved, as far as available, and information relevant to diagnostic classification was extracted at patient and study level, as appropriate.

### Major NCD

The diagnosis of major NCD is based on four main criteria, A–D, and two sub-criteria to criterion A (A1, A2). Criterion A requires a “significant cognitive decline from a previous level of performance in one or more cognitive domains”, evidence of which should be based on the two sub-criteria. A1 specifies that the “concern of the individual, a knowledgeable informant or the clinician” should be present; A2 “a substantial impairment in cognitive performance”. Both A1 and A2 must be met. Furthermore, the following are also mandatory: interference of cognitive deficits “with independence in everyday activities” (criterion B), occurrence not “exclusively in the context of a delirium” (criterion C) and that the “cognitive deficits are not better explained by another mental disorder” (criterion D).

In a substantial number of trials, the diagnosis of dementia, probable AD, probable VaD or possible AD with cerebrovascular disease (CVD) was established in accordance with DSM-III, DSM-III-R, ICD-10, NINCDS-ADRDA or NINDS-AIREN, as applicable. Therefore, the various sets of diagnostic criteria used in the included studies were compared to determine how and to what extent criteria of the DSM-5 correspond to criteria of the other classifications and whether fulfilment of DSM-5 criteria can be concluded from the fulfilment of corresponding criteria of other classifications. Additional information from inclusion and exclusion criteria, neuropsychological tests and rating scales supporting the diagnosis of major NCD was presented in the published reports on these trials.

If none of the sets of diagnostic criteria mentioned above was employed, all pertinent information, such as inclusion and exclusion criteria, means and distributions of actual test and rating scale scores, was utilized to check whether the criteria for major NCD were met.

### Mild NCD

The diagnosis of mild NCD is also based on four main criteria, A to D, and two sub-criteria to criterion A (A1, A2). Criterion A requires a “modest cognitive decline from a previous level of performance in one or more cognitive domains”, evidence of which should be derived from the two sub-criteria. A1 specifies that the “concern of the individual, a knowledgeable informant or the clinician” should be present; A2 “a modest impairment in cognitive performance”. Both A1 and A2 must be met. Moreover, it is mandatory that the cognitive deficits “do not interfere with capacity for independence in everyday activities” (criterion B), “do not occur exclusively in the context of a delirium” (criterion C) and are “not better explained by another mental disorder” (criterion D).

The consensus criteria for MCI [4] that were employed in one trial [14] explicitly cover criteria A (including A1 and A2) and B. From the exclusion criteria reported in the published paper, it is evident that criteria C and D were also met by the enrolled patients. The eligibility criteria of the other trials with patients who were cognitively impaired but not demented were as variable as the diagnostic terms assigned. Hence, all available information was utilized to check whether the criteria for mild NCD were met.

### Aetiological sub-classes

Testing for AD genetic mutations or genetically determined vascular disorders was not performed in any of the studies, nor was a specific family history documented. Hence, the classification of NCD due to probable or possible AD was mainly based on the course of the disease [“insidious onset and gradual progression” (criteria B and C2b), “clear evidence of decline in memory and learning” (criteria B and C2a)], lacking evidence of mixed aetiology (criterion C2c) and the fact that the disturbance is not better explained by another disorder (criterion D).

The classification of vascular NCD mainly relies on the temporal relationship of its onset to one or more cerebrovascular events (criterion B1) or evidence for a prominent decline in complex attention and frontal-executive function (criterion B2), evidence of the presence of cerebrovascular disease (criterion C) and the fact that the symptoms are not better explained by another disorder (criterion D). In the absence of genetic testing, the probability of vascular origin is assessed by neuroimaging and the temporal relationship with one or more cerebrovascular events.

The NINCDS-ADRDA criteria for probable Alzheimer’s disease cover the DSM-5 criteria A, B, C2 and D for major NCD due to probable Alzheimer’s disease. The NINDS-AIREN criteria cover the DSM-5 criteria A, B1, C and D and the probability criterion 2 for probable major vascular NCD. Possible AD (NINCDS-ADRDA) with CVD (NINDS-AIREN) can be classified under major NCD due to multiple aetiologies (specifically AD and CVD). In all trials that used the NINCDS-ADRDA and/or NINCDS-AIREN criteria, further information supporting the aetiological sub-classification was available from neuroimaging.

In studies not employing NINCDS-ADRDA and/or NINDS-AIREN criteria, all available information was utilized to check whether the DSM-5 criteria for aetiological sub-categories were met.

## Results

A total of 31 randomized controlled trials of *G. biloba* extract EGb 761^®^ in elderly patients with various degrees of cognitive impairment were identified. The inclusion diagnoses of 23 trials (74 %) could be classified as NCD. In four trials, the presence of NCD was likely, but could not be ascertained beyond doubt, and in four trials, the criteria for NCD were not met.

### Studies in major NCD

The diagnostic criteria for major NCD or dementia of the classifications used in the included trials are presented in Table 1, arranged in a way to highlight the features in common and the differences. Some salient differences are: (a) only DSM-5 specifies that “the individual, a knowledgeable informant or the clinician” has to show concern about the cognitive decline; (b) DSM-5 requires deficits in one or more domains, whereas all other sets of criteria require impairment in at least two or even more than two (NINDS-AIREN) domains; (c) DSM-5 is the only classification that allows a diagnosis of dementia without overt memory impairment—“unusual nonamnestic presentations… also exist”; (d) the NINCDS–ADRDA criteria do not explicitly mention interference with activities of daily living, but presence of dementia is required, with “dementia” usually being understood as cognitive decline severe enough to interfere with professional and/or social activities.

The DSM-5 criteria A2, B and C could be assumed to be met if any of the other classifications listed in Table 2 was used in a study. Fulfilment of criterion A1 was usually concluded from the patients seeing a clinician and undergoing examinations and diagnostic procedures. The DSM-III-R criteria for dementia in general do not explicitly require that there be a decline in cognitive abilities (criterion A), but the explanatory text mentions a loss in intellectual abilities and the criteria for both AD and MID require deterioration in cognitive performance in order to establish the diagnosis. The ICD-10 criterion G4, specifying that the cognitive decline should have been present for at least 6 months for a confident diagnosis of dementia, seems to have a similar intention, but is not explicit in saying that there should be no better explanation for the cognitive deficits than dementia. The ICD-10 criteria were used in one of the included studies, and the patients of this study also had to meet the DSM-III-R criteria. Overall, it can be concluded that all patients diagnosed with dementia using one of the sets of criteria listed in Table 1 can safely be classified as having major NCD in accordance with DSM-5.

**Table 2:** Studies of EGb 761^®^ that enrolled patients with major NCD and unambiguous aetiological sub-classification

Author (year of publication)	Diagnostic criteria used in the study	Tests for cognitive impairment and ADL/functional scales used to support diagnosis	DSM-5 diagnoses assigned retrospectively
Nikolova 2013 [21]	NINCDS-ADRDA, NINDS-AIREN	TE4D, SKT, VFT, CDT, GBS	Major NCD due to probable AD, probable vascular major NCD, major NCD due to multiple aetiologies (major NCD due to AD and vascular major NCD)
Herrschaft 2012 [22]	NINCDS-ADRDA, NINDS-AIREN	TE4D, SKT, VFT, CDT, ADL-IS	Major NCD due to probable AD, probable vascular major NCD, major NCD due to multiple aetiologies (major NCD due to AD and vascular major NCD)
Ihl 2011 [23]	NINCDS-ADRDA, NINDS-AIREN	TE4D, SKT, VFT, CDT, ADL-IS	Major NCD due to probable AD, probable vascular major NCD, major NCD due to multiple aetiologies (major NCD due to AD and vascular major NCD)
Napryeyenko 2007 [24]	NINCDS-ADRDA, NINDS-AIREN	TE4D, SKT, VFT, CDT, GBS	Major NCD due to probable AD, probable vascular major NCD, major NCD due to multiple aetiologies (major NCD due to AD and vascular major NCD)
Yancheva 2009 [25]	NINCDS-ADRDA	TE4D, SKT, VFT, CDT, GBS	Major NCD due to probable AD
Schneider 2005 [26]	NINCDS-ADRDA	MMSE, ADAS-cog, GERRI, PDS	Major NCD due to probable AD
Maurer 1997 [27]	NINCDS-ADRDA, DSM-III-R	BCRS, SKT, ADAS-cog	Major NCD due to probable AD
Le Bars 1997 [28]	DSM-III-R, ICD-10	MMSE, ADAS-cog, GERRI	Major NCD due to probable AD, probable vascular major NCD
Kanowski 1996 [29]	DSM-III-R	MMSE, SKT, NAB	Major NCD due to probable AD, probable vascular major NCD

*ADAS-cog* Alzheimer’s Disease Assessment Scale-cognitive subscale, *ADL-IS* Activities of Daily Living International Scale, *BCRS* Brief Cognitive Rating Scale, *CDT* Clock-Drawing Test, *GBS* Gottfries-Bråne-Steen Scale, *GERRI* Geriatric Evaluation by Relative’s Rating Instrument, *MMSE* Mini-Mental State Examination; *PDS* Progressive Deterioration Scale, *SKT* Syndrom-Kurztest (Short Cognitive Performance Test), *TE4D* Test for the Early Diagnosis of Dementia with Differentiation from Depression, *VFT* Verbal Fluency Test

Thirteen trials (42 %) enrolled patients that could be classified unambiguously as having major NCD. In nine of these trials the aetiological sub-classifications also meet the criteria set by DSM-5 (Table 2), in three trials the aetiological sub-classification could not be confirmed beyond doubt, and in one trial no sub-classification was made (Table 3). If more than one aetiological category was acceptable for a trial, the aetiological sub-classification was always made prospectively.

**Table 3:** Studies of EGb 761^®^ that enrolled patients with major NCD, aetiological sub-classification not entirely certain or not made

Author (year of publication)	Diagnostic criteria used in the study	Tests for cognitive impairment and ADL/functional scales used to support diagnosis	DSM-5 diagnoses assigned retrospectively
Rai 1991 [30]	NINCDS-ADRDA	MMSE, Kendrick battery for the detection of dementia	Major NCD due to AD^a^
Haase 1996 [31]	DSM-III-R	MMSE, GDS, KAI, NAB, NAA	Major NCD due to probable AD, possible vascular major NCD
Weitbrecht and Jansen 1986 [32]	Not specified	WAIS digit symbol substitution test, WAIS digit span test, SCAG, Crichton Geriatric Scale	Major NCD (most likely due to AD)^b^
Oswald 1997 [33]	DSM-III	SCAG, NAI	Major NCD (no aetiological sub-classification was made)

^a^Patients were diagnosed in accordance with NINCDS-ADRDA criteria, but the authors did not specify whether probable AD or possible AD or both were accepted

^b^The inclusion diagnosis assigned by the clinician was “primary degenerative dementia”, which was then used synonymously with dementia of the Alzheimer type, but diagnostic criteria were not specified. Hence, not all DSM-5 criteria for aetiology could be verified. The acceptable range for the Hachinski Ischaemic Score (up to 7) supports the clinical diagnosis of Alzheimer’s disease, but does not strictly exclude mixed AD/vascular aetiology

*GDS* Global Deterioration Scale, *KAI* Kurztest für Allgemeine Intelligenz (Short Test for General Intelligence), *MMSE* Mini-Mental State Examination, *NAA* Nürnberger Alters-Alltags-Skala (Nuremberg Gerontopsychological Self-Rating Scale for Activities of Daily Living), *NAB* Nürnberger Alters-Beobachtungs-Skala (Nuremberg Gerontopsychological Observation Scale for Activities of Daily Living), *NAI* Nürnberger Alters-Inventar (Nuremberg Gerontopsychological Inventory), *SCAG* Sandoz Clinical Assessment-Geriatric, *WAIS* Wechsler Adult Intelligence Scale

### Studies in major or mild NCD

Six trials (19 %) enrolled patients who could be classified unambiguously as having NCD, but interference of the cognitive deficits with independence in everyday activities (criterion B) was not completely clear (Table 4). Hence, no strict distinction between mild NCD and major NCD can be made. In two of these studies, the DSM-5 criteria for possible vascular NCD are fulfilled.

**Table 4:** Studies of EGb 761^®^ that accepted patients with NCD without clear distinction between major NCD and mild NCD

Author (year of publication)	DSM-5 diagnoses
Gräßel 1992 [34]	Possible vascular major NCD, possible vascular mild NCD
Halama 1988 [35]	Possible vascular major NCD, possible vascular mild NCD
Hofferberth 1994 [36]	Major NCD, mild NCD (aetiological sub-classification uncertain)^a^
Schubert and Halama 1993 [37]	Major NCD, mild NCD (no aetiological sub-classification was made)
Israël 1987 [38]	Major NCD, mild NCD (no aetiological sub-classification was made)
Wesnes 1987 [39]	Major NCD, mild NCD (no aetiological sub-classification was made)

^a^The inclusion diagnosis assigned by the clinician was “senile dementia of the Alzheimer type”, but diagnostic criteria are not specified. Hence, not all DSM-5 criteria could be verified. According to inclusion criteria and data recorded at baseline, more than 75 % of patients had major NCD, for 25 % this could not be verified beyond doubt. Cognitive deficits, Hachinski Ischaemic Score and CT scan support the clinical diagnosis of Alzheimer’s disease

### Studies in mild NCD

Patients meeting DSM-5 criteria for mild NCD were enrolled in four trials (13 %) (Table 5). No aetiological sub-classification was made in these trials.

**Table 5:** Studies of EGb 761^®^ that enrolled only patients with mild NCD

Author (year of publication)	DSM-5 diagnoses
Gavrilova 2014 [14]	Mild NCD (no aetiological sub-classification was made)
Grass-Kapanke 2011 [40]	Mild NCD (no aetiological sub-classification was made)
Allain 1993 [41]	Mild NCD (no aetiological sub-classification was made)
Stocksmeier and Eberlein 1992 [42]	Mild NCD (no aetiological sub-classification was made)

### Studies in patients not classified by DSM-5

Eight studies (26 %) enrolled patients who could not be classified unambiguously as having NCD in accordance with DSM-5 diagnostic criteria (Table 6). This does not necessarily mean that these patients did not have NCD; it only means that the information provided was not sufficient to safely conclude that all included patients had NCD.

**Table 6:** Studies of EGb 761^®^ enrolling patients not classifiable by DSM-5

Author (year of publication)	Original diagnosis
van Dongen 2003 [43]	Age-associated memory impairment (AAMI), dementia^a^
Hofferberth 1991 [44]	Organic brain syndrome with increased vascular risk
Halama 1990 [45]	Cerebrovascular insufficiency
Hofferberth 1989 [46]	Organic brain syndrome
Taillandier 1986 [47]	Chronic cerebral insufficiency
Eckmann and Schlag 1982 [48]	Cerebrovascular insufficiency
Dieli 1981 [49]	Chronic cerebral insufficiency
Moreau 1975 [50]	Chronic insufficiency of cerebral circulation

^a^The main inclusion diagnosis was AAMI, which could be mild NCD; a small proportion of patients were assigned a diagnosis of dementia applying DSM-III-R and ICD-10 criteria by nursing home staff not trained to diagnose dementia. The SKT scores were in the range typical for mild-to-moderate dementia, which renders the high rate of AAMI questionable. On the other hand, interference with activities of everyday life was denied for most patients. In the absence of laboratory tests and neuroimaging, the possibility of conditions other than AAMI or dementia must be taken into account and any classification in terms of NCD would be imprudent

## Discussion

Using available diagnostic and other descriptive information, predominantly from published papers, the patients of most (74 %) randomized controlled trials of *G. biloba* extract EGb 761^®^ could be classified in terms of the new DSM-5 diagnostic categories. As expected, a high proportion of trials in major NCD (nine out of 13) allowed an unambiguous aetiological sub-classification at the “probable” level (Table 2); in three trials the level of certainty was lower than “probable”, and only one trial in major NCD did not classify the patients by aetiology (Table 3). In contrast, no aetiological sub-classification was made in the four trials enrolling only patients with mild NCD (Table 5). This reflects the presence of aetiological sub-classes for dementia, but not for pre-dementia stages of cognitive impairment, in earlier classifications of mental disorders. For two of the six trials that admitted both major NCD and mild NCD, an unambiguous classification as vascular NCD was possible, and for another one a classification as NCD due to AD was reasonably likely.

Using mainly inclusion and exclusion criteria and severity information at study level for classification, a patient sample was classified under mild or major NCD and under an aetiological sub-group only if all patients met the respective criteria. Our procedure can therefore be regarded as conservative. If the inclusion diagnosis in terms of mild or major NCD or aetiology could not be ascertained for the whole study sample, this study population remained unclassified with respect to mild or major NCD (Table 4) or with respect to aetiology (Table 3).

The new DSM-5 concept integrates the early (pre-dementia) and later (dementia) stages of aetiologically different neurocognitive disorders with the common core feature “cognitive decline” within one common framework. The studies that admitted both patients with mild NCD and those with major NCD reflect a problem that has not been resolved completely by the DSM-5, i.e. a precise definition and operationalization of interference of the cognitive deficits with independence in everyday activities. Since the 1980s, the distinction between MCI and dementia or mild NCD and major NCD has been refined considerably, yet room for interpretation still remains. Interestingly, the Herbal Medicinal Products Committee (HMPC) of the European Medicines Agency (EMA) overcame this problem by phrasing the therapeutic indication for well-established use of *G. biloba* leaf extract as follows: “improvement of (age-associated) cognitive impairment and of quality of life in mild dementia” [15]. It thus acknowledges that there is evidence of efficacy from randomized controlled trials in both MCI and dementia or, in other words, mild NCD and major NCD.

By re-grouping patient samples of older studies in terms of DSM-5 diagnostic categories, well-defined and widely accepted diagnoses could be assigned to the patients of a considerable number of clinical trials in which old-fashioned and vaguely defined diagnostic terms were originally used. This does not immediately change the overall knowledge base concerning the efficacy of EGb 761^®^, but it enables comparisons between studies of different periods in time and appropriate aggregation of trials for systematic reviews and meta-analyses. As recently accomplished for dementia [16], a meta-analysis can now be conducted across studies of patients with mild NCD. The new classification also helps younger clinicians and researchers understand which types of patients were enrolled in studies conducted more than two decades ago.

Interestingly, a comparison of various sets of diagnostic criteria for dementia and major NCD found a high concordance, allowing a DSM-5 diagnosis of major NCD in a high proportion of patients who were diagnosed with dementia according to the older classifications. There is, however, no complete congruence between the classifications, with DSM-5 tending to be more inclusive than earlier classifications. Patients diagnosed, e.g. as having major NCD due to probable AD or probable vascular major NCD by DSM-5, would not necessarily meet the more elaborate NINCDS-ADRDA criteria for probable AD or NINDS-AIREN criteria for probable VaD.

Using the DSM-5 diagnostic term “neurocognitive disorder”, where appropriate, helps overcome some of the problems associated with the use of terms such as “dementia” or (cerebral, cerebrovascular) “insufficiency”, which are widely perceived as stigmatizing and devaluing [13, 17]. First, dementia (defined as deterioration or loss of the intellectual faculties, the reasoning power, the memory, and the will [18]) is an inappropriate term for the early stages of NCDs during which full independence or at least a large capacity to manage everyday life is retained. Second, a considerable number of family physicians are still reluctant to diagnose dementia and to communicate this diagnosis to patients and their families, due to the stigma associated not only with the disease itself, but with the term dementia in particular [17, 19]. On the other hand, the change in terminology implemented by psychiatrists, but not neurologists (e.g. NINCDS-ADRDA revision [11]; NIA-AA criteria [20]) may render communication and collaboration of neurologists and psychiatrists in the field of neurodegenerative diseases more difficult.

It is a particular strength of our research that DSM-5 diagnoses were assigned only if the available information permitted the verification of all pertinent diagnostic criteria for the whole patient sample. This means that the patients classified retrospectively met the same criteria as those that patients enrolled in clinical trials today are required to meet. A limitation is the retrospective approach, because the interpretation of today’s DSM-5 criteria may not be exactly the same as the interpretation of corresponding inclusion criteria stipulated 20 years ago. Moreover, increased awareness of the risk of developing NCD and refined diagnostic procedures may lead to higher proportions of very mildly affected patients in today’s specialized centres that recruit patients to clinical trials. However, similar variability between patient samples from different trials is to be expected even in the case of prospective diagnosis. This is likely to result from differences in the types of patients available at different clinical sites and the possibility of some diversity in interpretation of the diagnostic criteria in various regions with different cultures.

In conclusion, the new classification and nomenclature of the DSM-5 could be applied successfully to assign modern, consistent and meaningful diagnostic terms to elderly patients with cognitive impairments enrolled in clinical trials over the past two and a half decades. The patients of 74 % of the randomized controlled trials of *G. biloba* extract EGb 761^®^ identified by a comprehensive literature search could be retrospectively classified by matching selection criteria and baseline data to the DSM-5 diagnostic criteria.

## Electronic supplementary material

Below is the link to the electronic supplementary material.

Supplementary material 1 (DOCX 21 kb)
