# Supplementary material for: A retrospective classification of diagnoses in terms of DSM-5 for patients included in randomized controlled trials of Ginkgo biloba extract EGb 761®
Source: Eur Arch Psychiatry Clin Neurosci. 2015 Aug 13;266:249–59. doi: 10.1007/s00406-015-0632-y (PMC4819753; doi:10.1007/s00406-015-0632-y)
Supplement: Supplementary file 1 — Supplementary material 1 (DOCX 21 kb) [file 406_2015_632_MOESM1_ESM.docx]

Table e1: Checklist DSM-5 Major NCD

|  | A. Evidence of significant cognitive decline | | B. Cognitive deficits interfere with independence in everyday activities | C. Cognitive deficits do not occur exclusively in the context of a delirium | D. Cognitive deficits are not better explained by another mental disorder |
| --- | --- | --- | --- | --- | --- |
|  | A.1. concern of individual, knowledgeable informant or clinician | A.2. substantial impairment in cognitive performance |  |  |  |
| Nikolova 2013 [21] | concluded from seeing a clinician and undergoing diagnostic procedures | NINCDS-ADRDA/ NINDS-AIREN, TE4D≤35, SKT 9-23 | NINCDS-ADRDA/ NINDS-AIREN | NINCDS-ADRDA/ NINDS-AIREN, and exclusion criteria | NINCDS-ADRDA/ NINDS-AIREN, exclusion criteria, CT/MRI, labs |
| Herrschaft 2012 [22] |  | NINCDS-ADRDA/ NINDS-AIREN, TE4D≤35, SKT 9-23 | NINCDS-ADRDA/ NINDS-AIREN, supported by ADL-IS | NINCDS-ADRDA/ NINDS-AIREN, exclusion criteria | NINCDS-ADRDA/ NINDS-AIREN, exclusion criteria, CT/MRI, labs |
| Ihl 2011 [23] |  | NINCDS-ADRDA/ NINDS-AIREN, TE4D≤35, SKT 9-23 | NINCDS-ADRDA/ NINDS-AIREN, supported by ADL-IS | NINCDS-ADRDA/ NINDS-AIREN, exclusion criteria | NINCDS-ADRDA/ NINDS-AIREN, exclusion criteria, CT/MRI, labs |
| Napryeyenko 2007 [24] |  | NINCDS-ADRDA/ NINDS-AIREN, TE4D≤35, SKT 9-23 | NINCDS-ADRDA/ NINDS-AIREN | NINCDS-ADRDA/ NINDS-AIREN, exclusion criteria | NINCDS-ADRDA/ NINDS-AIREN, exclusion criteria, CT/MRI, labs |
| Yancheva 2009 [25] |  | NINCDS-ADRDA, TE4D≤35, SKT 9-23 | NINCDS-ADRDA | NINCDS-ADRDA, exclusion criteria | NINCDS-ADRDA, exclusion criteria, CT/MRI, labs |
| Schneider 2005 [26] |  | NINCDS-ADRDA, MMSE 10-24 | NINCDS-ADRDA | NINCDS-ADRDA, exclusion criteria | NINCDS-ADRDA, exclusion criteria, CT/MRI, labs |
| Maurer 1997 [27] |  | NINCDS-ADRDA, DSM-III-R, BCRS mean 3-5, ADAS-cog 9-52 | NINCDS-ADRDA, DSM-III-R | NINCDS-ADRDA, DSM-III-R, exclusion criteria | NINCDS-ADRDA, DSM-III-R, exclusion criteria, CT |
| Le Bars 1997 [28] |  | DSM-III-R, ICD-10, MMSE 9-26, GDS 3-6 | DSM-III-R, ICD-10, GDS 3-6 | DSM-III-R, ICD-10, exclusion criteria | DSM-III-R, ICD-10, exclusion criteria, CT/MRI |
| Oswald 1997 [33] |  | DSM-III, average of SCAG items 2, 3, 6 and 8 at least 3.5 | DSM-III | DSM-III, exclusion criteria | DSM-III, exclusion criteria |
| Kanowski 1996 [29] |  | DSM-III-R, MMSE 13-25 | DSM-III-R | DSM-III-R, exclusion criteria | DSM-III-R, exclusion criteria, CT |
| Haase 1996 [31] |  | DSM-III-R, GDS 4-5 | DSM-III-R, GDS 4-5 | DSM-III-R, exclusion criteria | DSM-III-R, exclusion criteria |
| Rai 1991 [30] |  | NINCDS-ADRDA, clinical assessment | NINCDS-ADRDA | NINCDS-ADRDA | NINCDS-ADRDA |
| Weitbrecht & Jansen 1986 [32] |  | required by inclusion criteria, verified by various tests and scales | required by inclusion criteria, supported by SCAG and Crichton Geriatric Rating Scale | exclusion criteria: other impairment of cerebral function | exclusion criteria: other impairment of cerebral function |

Table e2: Checklist DSM-5 Mild NCD

|  | A. Evidence of modest cognitive decline | | B. Cognitive deficits do not interfere with capacity for independence in everyday activities | C. Cognitive deficits do not occur exclusively in the context of a delirium | D. Cognitive deficits are not better explained by another mental disorder |
| --- | --- | --- | --- | --- | --- |
|  | A.1. concern of individual, knowledgeable informant or clinician | A.2. modest impairment in cognitive performance |  |  |  |
| Gavrilova 2014 [14] | Winblad consensus criteria [4] | CAMCOG<10^th^ percentile for age, sex and educational level | IQCODE mean score <4 | exclusion criteria: any other cognitive or mental disorder | exclusion criteria: any other cognitive or mental disorder, MRI scan |
| Grass-Kapanke 2011 [40] | subjective complaint of cognitive impairment, perceived as decline from former level of functioning | at least one standard deviation below normative group in at least one cognitive test | inclusion criterion: intact activities of daily living, non-demented | exclusion criteria: any other cognitive or mental disorder | exclusion criteria: any other cognitive or mental disorder |
| Allain 1993 [41] | self-reported, perceived cognitive impairment | more than one standard deviation below young persons in word list recall test | in-/exclusion criteria, non-demented (supported by MMSE 27-28) | in-/exclusion criteria: any other cognitive or mental disorder | in-/exclusion criteria: any other cognitive or mental disorder |
| Stocksmeier & Eberlein 1992 [42] | self-reported, perceived cognitive impairment | below 16^th^ percentile in test for concentration and memory | in-/exclusion criteria, non-demented | in-/exclusion criteria: any other significant cognitive or mental disorder | in-/exclusion criteria: any other significant cognitive or mental disorder |

Table e3: Checklist DSM-5 NCD without clear distinction between mild and moderate NCD (*criteria that may indicate mild or major NCD printed in italics*)

|  | *A. Evidence of modest or significant cognitive decline* | | *B. Cognitive deficits do not interfere with capacity for independence in everyday activities* | C. Cognitive deficits do not occur exclusively in the context of a delirium | D. Cognitive deficits are not better explained by another mental disorder |
| --- | --- | --- | --- | --- | --- |
|  | A.1. concern of individual, knowledgeable informant or clinician | *A.2. modest or substantial impairment in cognitive performance* |  |  |  |
| Gräßel 1992 [34] | concluded from seeing a clinician and undergoing diagnostic procedures | *cognitive decline, i.e. current IQ (derived from short term storage capacity) < pre-morbid IQ (derived from verbal intelligence test)* | *no criterion* | exclusion criteria: any other neurological or psychiatric disorder | exclusion criteria: any other neurological or psychiatric disorder |
| Halama 1988 [35] | concluded from seeing a clinician and undergoing diagnostic procedures | *cognitive impairment documented by cognitive testing and rating scales* | *no criterion* | exclusion criteria: other significant disorders | exclusion criteria: other possible reasons for cognitive impairment |
| Hofferberth 1994 [36] | concluded from seeing a clinician and undergoing diagnostic procedures | *Blessed Dementia Rating Scale Part A: 0 – 16 and Part B 9.5 – 30.5; SKT scores supportive of dementia* | *no criterion* | exclusion criteria: other neurological or psychiatric disorders, pronounced sensory or motor disturbances | exclusion criteria: other neurological or psychiatric disorders, intellectual deterioration of other origin |
| Schubert & Halama 1993 [37] | concluded from seeing a clinician and undergoing diagnostic procedures | *at least 10 points on C.I. Scale* | *no criterion* | exclusion criteria: any other neurological or psychiatric disorder | exclusion criteria: any other neurological or psychiatric disorder |
| Israël 1987 [38] | self-reported memory complaints | *MMSE 20-26* | *no criterion* | exclusion criteria: any significant acute or chronic disease | exclusion criteria: any significant acute or chronic disease |
| Wesnes 1987 [39] | concluded from seeing a clinician and undergoing diagnostic procedures | *Crichton Geriatric Behavioural Rating Scale at least 14, but no item rated higher than 3* | *"mild impairment of everyday functioning" was required, but no clear statement of influence on idependence* | exclusion criteria: any neurological or psychiatric disorder that could cause cognitive impairment | exclusion criteria: any neurological or psychiatric disorder that could cause cognitive impairment |
